# Simple and Precise Approach for Determination of Ohmic Contribution of Diaphragms in Alkaline Water Electrolysis

**DOI:** 10.3390/membranes9100129

**Published:** 2019-10-04

**Authors:** Jesús Rodríguez, Simonetta Palmas, Margarita Sánchez-Molina, Ernesto Amores, Laura Mais, Roberto Campana

**Affiliations:** 1 Centro Nacional del Hidrógeno (CNH2). Prolongación Fernando El Santo s/n, 13500 Puertollano, Ciudad Real, Spain; margarita.sanchez@cnh2.es (M.S.-M.); ernesto.amores@cnh2.es (E.A.); roberto.campana@cnh2.es (R.C.); 2 Dipartimento di Ingegneria Meccanica, Chimica e dei Materiali, Università degli studi di Cagliari, Via Marengo 2, 09123 Cagliari, Italy; segramm@dimcm.unica.it (S.P.); l.mais@dimcm.unica.it (L.M.)

**Keywords:** alkaline water electrolysis, diaphragm, ionic conductivity, ohmic contribution

## Abstract

A simple and low-cost alternating current (AC)-based method, without electrolyte correction, is proposed (Electrochemical Impedance Spectroscopy (EIS)-Zero Gap Cell) for the determination of ohmic contribution of diaphragms. The effectiveness of the proposed methodology was evaluated by using a commercial Alkaline Water Electrolysis (AWE) diaphragm (Zirfon^®^). Furthermore, the results were compared with two conventional electrochemical methodologies for calculating the separator resistance, based on direct current (DC), and AC measurements, respectively. Compared with the previous techniques, the proposed approach reported more accurate and precise values of resistance for new and aged samples. Compared with the manufacturer reference, the obtained error values for new samples were 0.33%, 5.64%, and 41.7%, respectively for EIS-Zero gap cell, AC and DC methods, confirming the validity and convenience of the proposed technique.

## 1. Introduction

Hydrogen, as an energy carrier, represents a promising solution for problems related to current fuel-based energy system [1]. It provides a sustainable fuel for a wide range of applications, from transportation to small electronic devices or stationary applications. Among other methods, one way, if not the only way, to produce green H_2_ is water electrolysis, if electrical input is obtained from a renewable energy (RE) source. For this reason, in the past decades, great attention on combination RE-H_2_ technologies was paid by energy companies, to set themselves in the future markets of distributed power generation and alternative fuels [2].

From a technical point of view, different technologies may be considered to perform water electrolysis, such as: Alkaline (AWE), proton exchange membranes (PEMWE), and solid oxide (SOWE) electrolysis. Among them, AWE is considered an optimal solution for RE combination, because it is the most economical and mature technology for H_2_ electrochemical production, especially at a large scale [3]. In fact, the largest water electrolysis systems are, nowadays, alkaline electrolyzers. Currently, AWE systems of up to 6MW are commercially available [4].

As the energetic requirement for water electrolysis is considered, most of the energy input is due to the high activation losses of the reactions involved at the electrodes, as well as to ohmic losses related to bubbles formation and to the charge transfer process within the electrolyte and separator. However, due to the very high concentration of the electrolyte used in alkaline electrolysis (25–32% KOH w/w), ohmic drop through the separator represents one of the points of great concern [5]. It is worth noting the crucial role of separator, since it must ensure the separation between gases (to avoid explosive mixtures) and, at the same time, favor the OH^−^ ions transport. In this context, improving the ionic conductivity of the separator could be one of the possible strategies to make the whole process more efficient. 

Up to the end of the 20th century, asbestos was the most used material; however, its use was eventually forbidden by the European Commission because of its potential health risk [6]. Moreover, some authors reported the low chemical resistance of this material to strong alkaline media [7]. Candidates for asbestos substitution should have such suitable characteristics as: Low resistance, good mechanical stability, high corrosion resistance, long lifetime, low H_2_ crossover and, finally, being economically acceptable [8]. Different materials have been proposed and tested this aim, such as ion-exchange polymeric membranes, made on conductive materials and functionalized for separating ions from solution. The application of these membranes in AWE has not been successful, mainly due to the poor chemical stability of the materials in strong alkali media, but also to membrane fouling [9]. 

On the other hand, ceramic-based diaphragms are considered a better alternative [10,11]. Ceramic diaphragms are microporous components, made of non-conductor material, which should act as a physic barrier, allowing the charge transport through micro-channels and, in turn, the ionic conduction between cathodic and anodic chambers. Recently, the interest for ceramic diaphragms for AWE has increased, and different ceramic materials have been tested, such as: Alumina-Kaolin [9,10], Wollastinite, and Olivine [11], ZrO_2_ [12], etc. 

Considering the specific characteristics of diaphragms, determining their resistivity is not simple: Unlike ionic membranes, diaphragms are not functionalized, and their conductivity is only due to ions of electrolyte in the micro-channels. Calculating this parameter is more critical when working with very porous materials in high conducting media, because the ionic resistivity of the electrolyte and diaphragm could present low and very similar values. In this case, even small experimental errors can heavily affect the results.

Different methodologies were proposed in the literature to determine the resistivity of diaphragms [13,14,15,16]. Typically, in these techniques, the resistance of the separator is obtained as a difference between the resistances of the system, measured in the presence, and in absence of, the separator, respectively. However, since many experiments are required in this procedure, the correction could cause of increasing errors. Moreover, electrochemical methods for resistance determination vary depending on DC or AC measurements. AC techniques are expected to be more accurate, although expensive and more precise instrumentation is required. On the other hand, as far as we know, there are no proposed methodologies, which allow the direct calculation of the conductivity of channels for diaphragms. In the case of ion exchange membranes, ionic conductivity is usually calculated by direct determination methods, based on direct (DC) [9,13] or alternating current (AC) [14], with two [15] or four electrodes [16]. However, the application of these methodologies for diaphragms could be more difficult because of cell architecture or electrolyte retention limitations. 

Furthermore, to the best of our knowledge, there are no studies in the literature, which compare different electrochemical methods for calculating the resistance contribution of diaphragms in AWE applications. Then, the selection of one or another approach can be more influenced by the instrumentation availability, than technique accuracy. 

For these reasons, in the present work, we propose a simple approach to measure the ionic resistance contribution of diaphragms for AWE applications. An optimized technique has been designed to reduce the typical error sources identified in other methodologies. To this aim, the presented approach is based on:

- Reducing resistance sources: Direct contact between electrodes and diaphragm eliminates the resistance contribution of electrolyte in the bulk.
- Direct measurement: The studied approach is based on electrochemical methodologies for determining the ionic resistance of polymeric membranes. In these approaches, electrodes are in direct contact with the membrane.
- Electrochemical Impedance Spectroscopy (EIS) measurements: AC measurements are more accurate than DC ones. Because of this, EIS techniques are used instead of chronopotentiometry or other DC techniques.
- Easy technical approach: Cell and components are manufactured with cheap materials, resulting in an economical set-up.

Accuracy, reproducibility and precision of this simple method were tested by determining the resistivity of Zirfon^®^ (AGFA, Mortsel, Belgium), which is the conventional commercial material for AWE diaphragms. In order to evaluate the convenience of the proposed approach, the results were compared with those obtained in previous works, from typical methods based on DC and AC measurements. 

The methodology presented in this paper showed the best results in terms of higher precision and lower error.

## 2. Materials and Methods 

### 2.1. Zirfon Perl^®^ as Diaphragm for AWE

**Figure 1:**
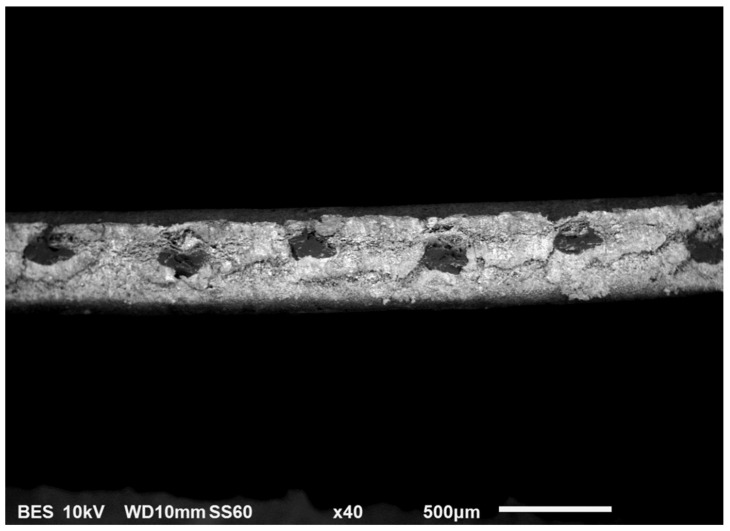
Cross section of a 500 µm thick Zirfon Perl 500 UTP^®^ separator.

Zirfon Perl^®^ (AGFA) was used as reference material. Zirfon^®^ is a porous composite diaphragm composed of a polysulfone network and ZrO_2_ as the inorganic filler [17]. In Figure 1, a scanning electron microscope (SEM) image of Zirfon Perl 500 UTP^®^ is shown. In this picture, the mesh polymeric fabric can be easily identified. The rest of the separator consists of a polymer-ZrO_2_ mixture coating. 

Zirfon^®^ contains 85 wt% of hydrophilic ZrO_2_ powder with a high specific surface area of 22 m^2^ g^−1^ as hydrophobic agent and 15 wt% polysulfone, which gives the material its mechanical strength [18]. Zirfon^®^ offers a high chemical stability in high KOH solutions, even at elevated temperatures. It also shows good structural stability and low resistance, leading to a significant improvement in the water electrolyzers’ performance [12]. In Table 1, the main characteristics of Zirfon^®^ are presented. 

### 2.2. Zirfon^®^ Characterization

Zirfon^®^ was morphologically characterized by Scanning Electron Microscopy (Jeol JSM 6010, JEOL, Ltd, Tokyo, Japan) and Electron Diffraction Scattering (EDS). Previously, Zirfon^®^ samples were coated with a thin carbon film by Thermal Evaporation of carbon to avoid misleading charging effects. 

AWE polarization curves, using Zirfon^®^ as diaphragm, were carried out in an electrochemical cell (MicroCell, Electrocell A/S, Tarm, Denmark) integrated in an AWE test bench designed and constructed by CNH2 (National Science Foundation, Alexandria, VA, USA) [1,3]. Current was applied by a power source (Elektro-Automatik EA-PSI 6000, EA Elektro-Automatik GmbH & Co.KG, Viersen, Germany), and both potential and current were monitored by a Supervisory Control and Data Acquisition (SCADA) system developed by CNH2. In the range 0–100 mA/cm^2^, current and voltage data were taken every 8-10 mA/cm^2^, while in the range 100–400 mA/cm^2^ were taken every 20–50 mA/cm^2^. Nickel 200 and Stainless Steel (SS316L) plate electrodes were used as anode, and cathode, respectively. Polarization curves were performed at ambient temperatures, using KOH (30 wt%) as electrolyte. Furthermore, long-time experiments were also done with a double scope: To analyze the performance of Zirfon^®^ after a long-time operation, and to test the proposed methodology for “aged samples”. In this case, as suggested in a previous work [1], ageing of samples was obtained by means of galvanostatic tests (at 200 mA/cm^2^ and 400 mA/cm^2^) at 60 °C, for 130 h.

### 2.3. Area Resistance Determination

The resistance of Zirfon^®^ was determined by a simple and cheap method, and the repeatability and accuracy of the measure was studied. The results have been compared with other two methodologies proposed by the literature. In all three cases, resistance was experimentally obtained and evaluated in terms of Area Resistance (AR, Ω·cm^2^), which is the value offered by the Zirfon^®^ manufacturer.

Area Resistance was calculated by Equation (1):

(1) $$\mathrm{AR}=A\cdot R_{D}$$

where A (cm^2^) is the apparent cross-sectional diaphragm’s surface and R_D_ (Ω) is diaphragm resistance.

The internal cell resistance (R_C_, Ω) is expressed as the sum of electrolyte (R_e_, Ω) and diaphragm (R_D,_ Ω) resistances:

(2) $$R_{C}=R_{e}+R_{D}$$

Thus, R_D_ can be experimentally calculated as the difference between cell resistance with, and without, a diaphragm.

R_D_ is related to geometrical factors as porosity (ε) and thickness (L, cm) [11]:

(3) $$R_{D}=\frac{l}{\kappa \cdot A_{p}}$$

(4) $$A_{p}=\varepsilon \cdot A$$

(5) l=L·τ

where l (cm) is the average channels length in the diaphragm, A_p_ (cm^2^) is the cross-sectional diaphragm’s surface, containing only pores, κ (Ω^−1^·cm^−1^), is the electrolyte conductivity and τ is the tortuosity. 

Based on Equations (3) and (5), Stojadinović et al. [11] proposed a procedure, which enables the calculation the ionic conductivity in microchannels of diaphragms (κ_D_, Ω^−1^·cm^−1^), considering the effect of geometrical parameters:

(6) $$R_{D}=\frac{L\cdot \tau }{\kappa \cdot \varepsilon \cdot A}$$

(7) $$\kappa_{D}=\kappa \cdot \frac{\varepsilon }{\tau }$$

Accordingly, this model allows the geometrical characterization of diaphragms (tortuosity) to be obtained by determining resistance and conductivity.

As explained below, R_D_ and R_e_ were calculated by DC, or AC methods, respectively. Depending onhe t specifications of each analyzed method, the subtraction of R_C_ and R_e_ is required. 

For all methods, two diaphragm samples were used to validate the measurements: An aged sample and a new sample. Three or four pieces of each type were analyzed, and each measure was repeated 5 times. Experimental error was calculated in terms of Standard Deviation (SD) and Coefficient of Variation (CV) [20]. Finally, for each method, the percent error was evaluated, with respect to the AR value supplied by the Zirfon^®^ manufacturer (AGFA, Mortsel, Belgium)

### 2.4. Methods for Calculation of Diaphragms Resistance 

A brief description of both methodologies proposed in literature is reported as follows. Finally, the method presented in this work is described. 

#### 2.4.1. Direct Current Measurement

**Figure 2:**
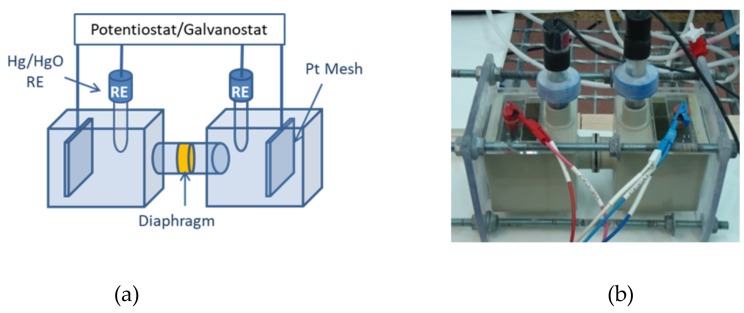
Direct Current Measurement Setup: (**a**) Scheme (**b**) Real cell.

As proposed by Agel et al. [12], this technique is based on a four-electrode potentiometric method. Experimental setup (Figure 2) consisted of a two-chamber electrochemical cell with two platinum mesh electrodes (Goodfellow Cambridge Ltd, Huntingdon, England) and two Hg/HgO reference electrodes (Koslow Scientific Company, Englewood, NJ, USA). 

**Figure 3:**
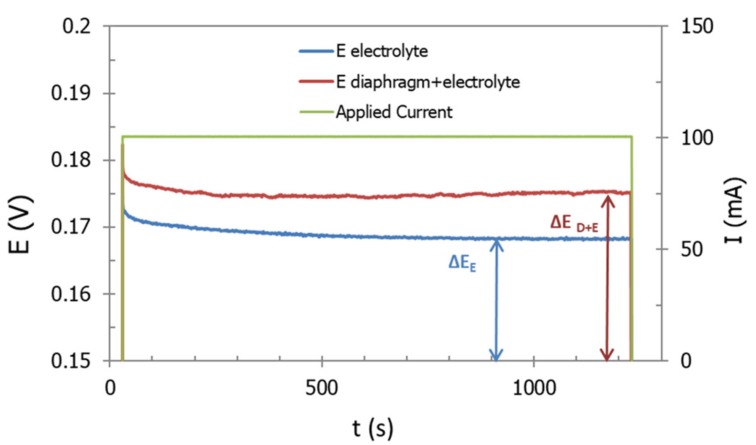
Example of trend in time of potential and current during a typical potentiometric measurement for resistance determination.

In a typical assay, a constant current was applied between Pt electrodes, when the two semi-cells were separated by the diaphragm: The current was applied for 20 min, up to a stable potential (E) value was reached (see Figure 3). Ohm’s law was then applied to calculate the resistance of the cell (R_D + El_, Ω), which in this case includes the resistances of the electrolyte (R_El_, Ω) and the diaphragm (R_D_). The same measure was repeated in the absence of diaphragm and, in this case, R_El_ was obtained. Finally, R_D_ was calculated by the difference between the two resistances above calculated. Potentiometric tests were carried out at lab temperature with a KOH (30 wt%) as electrolyte.

(8) $$R_{D}=R_{D+\mathrm{El}}-R_{\mathrm{El}}$$

(9) $$R_{D}=\frac{∆E_{D+\mathrm{El}}}{I}-\frac{∆E_{\mathrm{El}}}{I}$$

#### 2.4.2. EIS-Method

**Figure 4:**
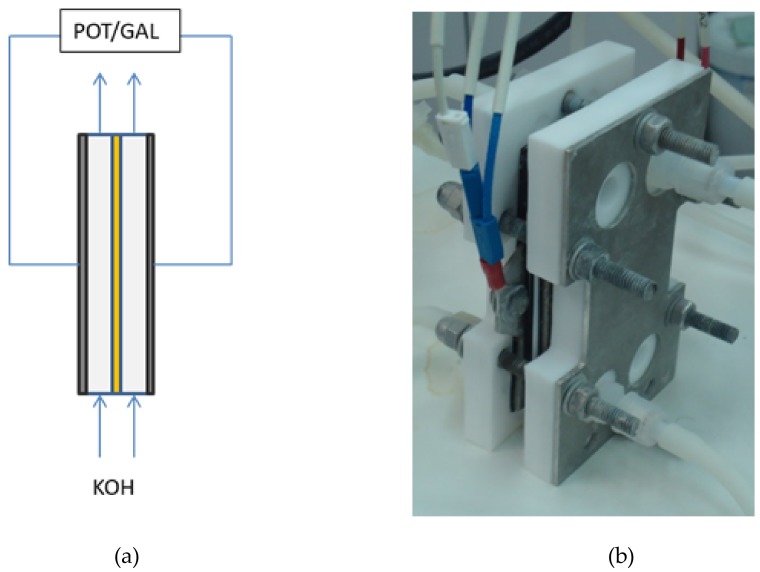
Electrochemical Impedance Spectroscopy (EIS)-Method Setup: (**a**) Scheme (**b**) Real cell.

According to the literature [7], Electrochemical Impedance Spectroscopy (EIS) represents a very effective method for resistance determination of diaphragms, due to a quite stable cell response, and a high precision measurement is obtained with this technique. In this case, a two-compartment electrochemical cell (MicroCell, ElectroCell Europe A/S, Tarm, Denmark), was used, connected to a multichannel 300V-Biologic Potentiostat/Galvanostat, equipped with a frequency response analyzer (FRA). Two SS316 electrodes acted as working and counter electrodes (Figure 4).

Following the methodology described by [7], EIS experiments were run in galvanostatic mode. A bias current of 100 mA was applied, to which a sinusoidal signal of 10 mA amplitude was superimposed. Impedance data were collected in the frequency range 100 kHz–3 Hz using six points per decade. The ohmic resistance of cell could be derived by the Nyquist plot (imaginary versus real components of impedance), from the real component of the impedance, measured as intercept of the curve at highest frequency value [7,21]. 

Where needed, equivalent circuit approach was used in order to interpret and quantify the EIS results. 

During a typical assay, the diaphragm samples were immersed in KOH (30 wt%) for 24 h. Afterwards, the EIS spectra were obtained with, and without, diaphragm. Each measurement was repeated 5 times, and the average value of resistance was obtained for assays with, and without, a diaphragm. The diaphragm’s resistance was obtained from subtracting the average values. 

#### 2.4.3. Zero-Gap EIS Method

On the bases of the typical techniques, used in Fuel Cell technology for anionic and cationic exchange membranes [21,22], in the present work, the Zero-Gap EIS Method is proposed. The main problem in case of diaphragms is that, in contrast with functionalized membranes, Zirfon^®^ is not ionic conductor at alkaline electrolysis operation temperatures. As explained above, conductivity of Zirfon^®^ depends on electrolyte channels and, to use the proposed methodology, the electrolyte should be efficiently retained inside the diaphragm. In this context, the challenge is to perform the measurement without immersing the electrodes and diaphragm in the electrolyte. To this aim, as suggested by other authors [21,22], a “sandwich cell” configuration can be adopted. However, two critical aspects have to be considered: Pre-treatment of the diaphragm in the electrolyte, and the pressure of terminal plates over the diaphragm, which is controlled by bolt torque. Pre-treatment usually consists in the immersion of diaphragms in KOH 30% at lab temperature for a certain time. The suitable immersion time was determined by immerging samples in KOH (30 wt%) at an ambient temperature for different times and measuring the variation of samples resistance. On the other hand, when the pressure at the terminal plates is concerned, if bolt torque is very high, electrolytes can flow out of the diaphragm. On the contrary, if bolt torque is not sufficiently high, problems related to poor electrode-diaphragm contact can occur. To determine an appropriate terminal plates strength, a bolt torque variation in the range of 0.5–1.5 N·m was applied by a torque wrench and the resistance of diaphragm samples was determined by EIS.

Once the suitable bolt torque and pre-treatment time were determined, the electrochemical measurements of resistance were carried out. Before measurements, both sides of samples surface were carefully dried with wet tissue paper, in order to remove the excess of KOH electrolyte and then the diaphragm was sandwiched between both electrodes. 

**Figure 5:**
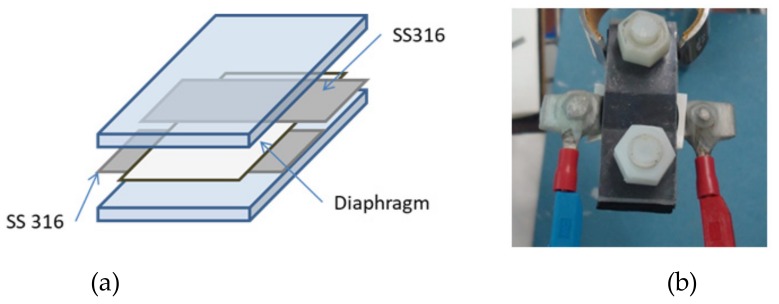
Zero-Gap EIS Method Setup: (**a**) Scheme (**b**) Real cell.

As in the previous method, the resistance of the membranes was determined by EIS. Two-point technique was employed using two stainless steel electrodes as Working Electrode, and Counter Electrode, respectively. The diaphragm was sandwiched between two electrodes, avoiding direct contact between them, and then introduced into the conductivity cell, according with Figure 5. Resistance measurements were immediately performed. 

**Figure 6:**
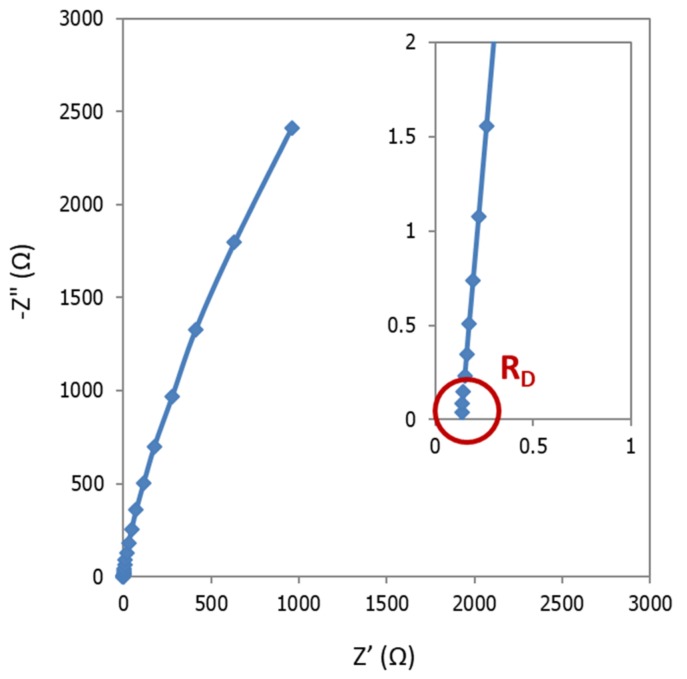
Nyquist plot obtained by Zero-Gap EIS Method. Inlet: high frequencies zone.

In this case, a potentiostatic method was preferred rather than galvanostatic, as some authors [11] suggest it can avoid the non-linear response of the system, that is frequently found at lower frequencies. The experiments were realized at open circuit potential, in order to avoid possible gas generation, which can cause high resistance zones, affecting the EIS response. The frequency was swept from 100 kHz to 3 Hz, with an AC signal amplitude of 10 mV around the open circuit potential, using six points per decade. As in the previous case, the diaphragm resistance could be obtained from the intercept of the real axis of the Nyquist plot at high frequencies (Figure 6). Area Resistance was directly calculated with Equation (1). 

## 3. Results and Discussion

**Figure 7:**
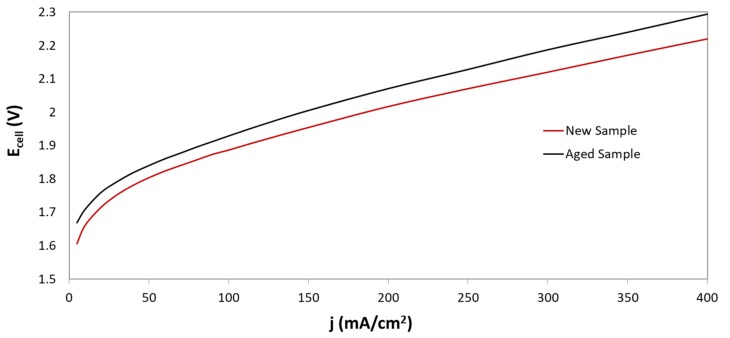
Polarization curves for electrolysis cells at T_amb_, KOH (30 wt%) for Zirfon^®^ before and after operating for 130 h (aged sample).

**Figure 8:**
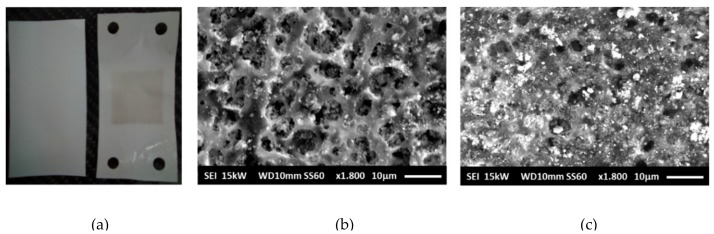
Picture of a new ((**a**)- left side), and 130h aged Zirfon^®^ sample ((**a**) – right side). SEM images for new (**b**), and 130 h aged (**c**) Zirfon^®^ samples.

During long operation, electrolysis cell progressively tends to reduce its performance, as can be observed in Figure 7. In fact, by comparing the response of the cell with new and aged samples, it can be seen that the deterioration of electrochemical performance is presented at both low, and high, current densities. For instance, for current densities values of 100 mA/cm^2^ and 400 mA/cm^2^, potential values of 1.88V, and 2.22 V were obtained for new samples. However, for aged samples, the potential achieved 1.93V, and 2.29 V, respectively. Although, the worsening of the electrochemical response could be caused by different processes, those occurring in the diaphragm play a significant role. The effect of ageing on the morphology of Zirfon^®^ diaphragms is shown in Figure 8. After long-time operation, the Zirfon^®^ samples showed a dark deposition over the surface area exposed to electrolyte (Figure 8). A brown-grey square, corresponding to the active area of the diaphragm (10 cm^2^), can be clearly identified (Figure 8a). SEM images for new and aged samples (Figure 8b,c, respectively) confirmed the formation of some deposits over the aged sample surface, which could partially or totally obstruct the available pores: Significant reduction of number of big pores is easily identified by comparing both images.

**Figure 9:**
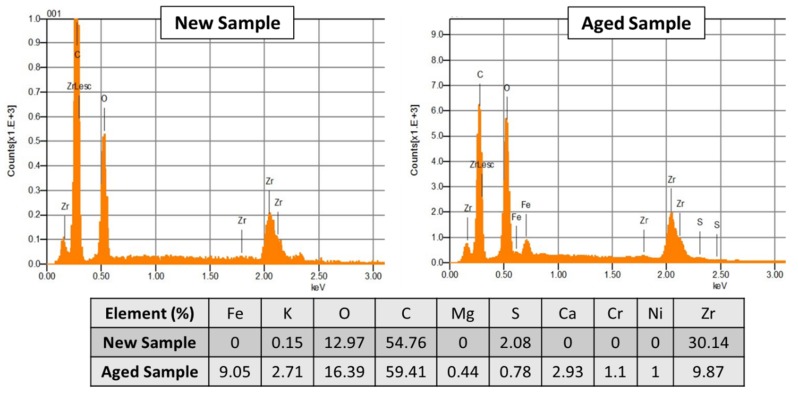
Electron Diffraction Scattering (EDS) Spectra and elemental composition for new and aged Zirfon^®^ samples.

EDS Spectra (Figure 9) demonstrated that the deposits mainly corresponded to iron compounds (with a percentage close to 9% of mass). The origin of these deposits could be the auxiliary equipment, such as tubing, adapters, pumps, liquid-gas separators, which under AWE operation conditions, could easily corrode. The covering of pores could result in a poorer charge transport, and then in an increase in ohmic resistance of the diaphragm. 

### 3.1. Direct Current Method

**Figure 10:**
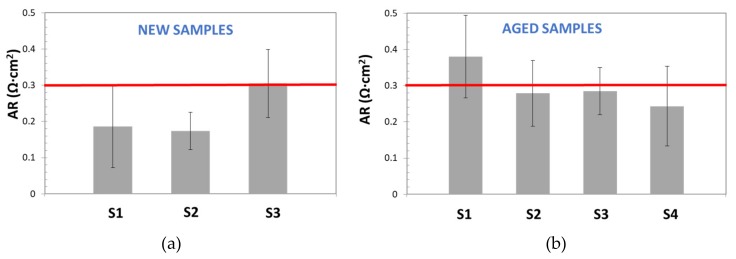
Area Resistance values, calculated for new (**a**) and aged (**b**) diaphragm samples. Each measurement was repeated 5 times (error bars are reported for each set of measurements).

In Figure 10, AR values, obtained by the DC method for new and aged samples, are reported and compared with the commercial reference value for Zirfon^®^ [18] (red line): ≤0.3 Ω cm^2^ (KOH 30 wt%, 30 °C). 

This figure demonstrates the low repeatability and the poor accuracy of the DC Method. On one hand, for both, new and aged samples, most of calculated AR values were far from the reference one; on the other hand, wide standard deviation bars were obtained (sometimes higher than 20%), even for the sample with AR similar to the reference. Although, standard deviation variation seems too high, it is in good agreement with the results observed by other authors. For instance, Agel et al. [13] obtained SD values in the range of 10–20%, for anionic membranes in KOH, at a concentration varying between 0.1 and 7.2 M. 

### 3.2. EIS Method

**Figure 11:**
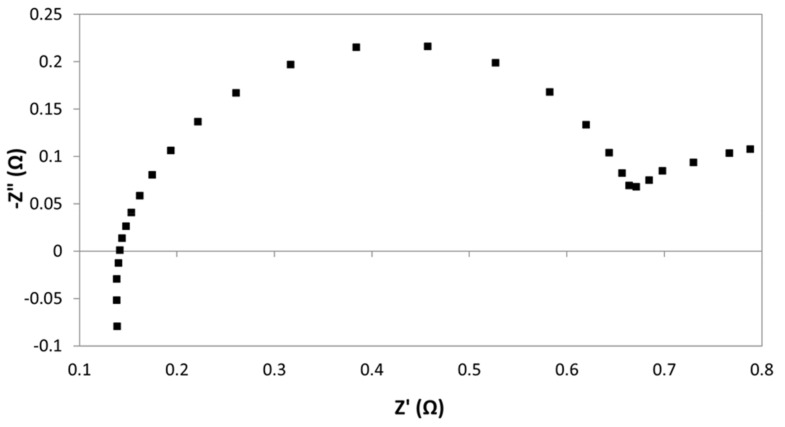
Example of the results from EIS measurements of diaphragm resistance.

As we can observe from Figure 11, parasitic phenomena were identified at high frequency in the fourth quadrant of the Nyquist, which were attributed to inductance effects caused by possible wires connections between potentiostat and electrochemical cell. According to the bibliography [23,24], while capacitance effects may dominate in the case of large systems with high impedance, inductance may dominate the errors in low impedance systems, such as in the present study [24]. In fact, inductance is one of the most common interfering factors when measuring through-plane impedance [22]: Configuration of the cell, but specially length and nature of electrical wires can strongly determine inductance effects [24]. 

Notably, when working with very concentrated electrolytes, as in the present case, the difference between the resistance of diaphragm and electrolyte is very low, so that, even small perturbations caused by inductance can induce high errors. Thus, the correction of inductance contribution becomes of paramount importance. 

As reported by other authors, this can be done by short circuit calibration procedures [23,24]. Firstly, impedance of the shorted cell is measured under the same conditions as those used for diaphragms measurements. Then, correction of the impedance is done at each frequency, under the assumption that the impedances of the diaphragm and the parasitic components are additive quantities. 

**Figure 12:**
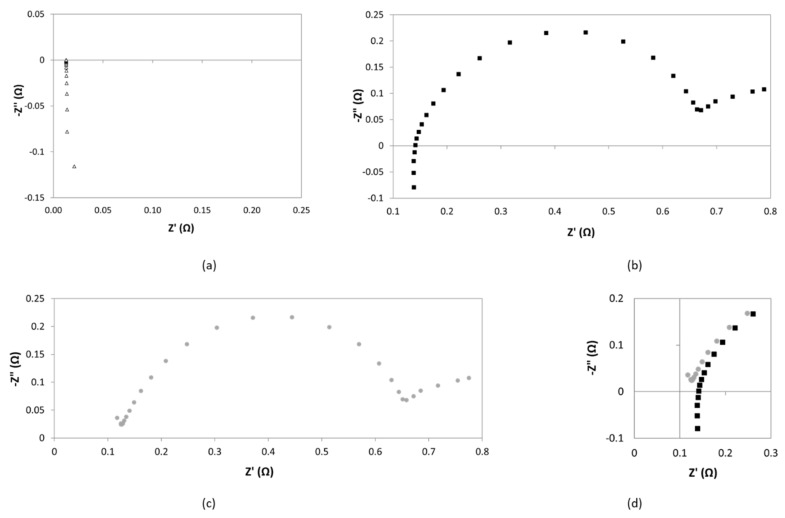
Correction procedure. Nyquist plots of (**a**) short circuit, (**b**) experimental data without correction, (**c**) experimental data after correction with inductance and (**d**) high frequency zone before and after correction.

An example of the data obtained by this procedure is reported in Figure 12. In Figure 12a, it is shown the Nyquist diagram in short circuit conditions. It exhibits a common pipe shaped form, which indicates that the shift of inductance from the zero point determines the internal resistance of the cabling [25,26]. Figure 12b reports the EIS response at conditions as detailed Section 2.4.2. In Figure 12c it is presented the correction of experimental data following the current approach. Finally, Figure 12d corresponds to the comparison between (b) and (c) at high frequencies zone. From this region, resistance of the cell can be obtained. 

After correction, curve intersection with real axis shifts towards smaller values. This confirms that the correction allows a high accurate determination of separator resistance. However, as in this example, in most of the measurements, the resulting corrected diagram does not properly intercept the real axis. Thus, for accuracy purposes, the determination of the resistance of diaphragm was done by means of the equivalent circuit approach. 

**Figure 13:**
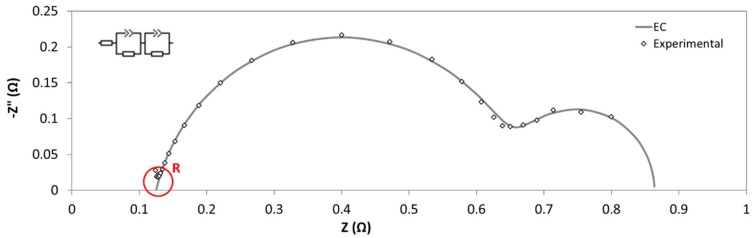
Nyquist diagram for determination of diaphragm resistance. Inlet: equivalent circuit used for determination of resistance value.

A typical Nyquist plot is shown in Figure 13. Among with the EIS results, it is also presented the fitting data, calculated by using the equivalent circuit reported in the inset of this figure. In particular, the circuit consisted of two constant phase element (CPE)-Resistance parallel elements, and one series resistance. CPE instead of capacitance were needed to take into account for non-ideal behavior of capacitors [27]. Each one of CPE-Resistance elements represents the electrochemical reaction at cathode, and anode, respectively. Series resistance corresponded to ohmic losses: Diaphragm, wires, electric contacts, electrolyte, etc. This resistance coincides with real impedance part at high frequencies.

From subsequent assays, with and without diaphragm and using Equations (1) and (8) the Area Resistance of diaphragm was obtained.

**Figure 14:**
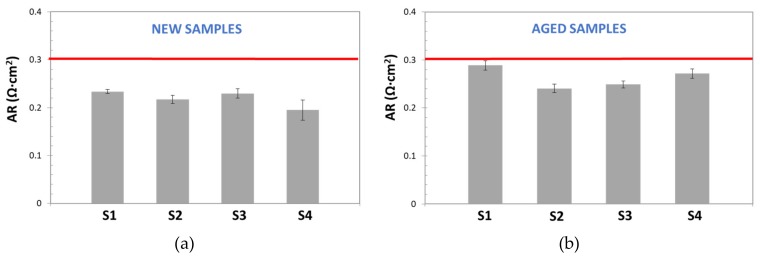
Area Resistance values calculated by EIS Method for: (**a**) new diaphragm samples (**b**) Used diaphragms.

Once the EIS response was corrected, AR was determined for Zirfon^®^ diaphragms, as described in Section 2. Figure 14 shows the results obtained for new and aged samples. It is possible to confirm that more stable area resistance values were obtained by this technique. The results also evidenced that EIS-based methods (Alternating Current) offered more precise and repeatable measurements than Direct Current techniques, and so it makes them more appropriate for electrochemical characterization of low resistance components. Furthermore, low standard deviation values were calculated for all studied samples. For new samples SD was in the range of 7.4–9.7 × 10^−4^ Ω, while for aged samples it varied from to 4 × 10^−4^ to 2 × 10^−2^ Ω. These values represent a significant increment of precision respect the DC method. 

However, the results for new and aged samples were always lower than area resistance values reported by the manufacturer (≥0.3 Ω·cm^2^): 0.19–0.24 Ω·cm^2^ and 0.24–0.29 Ω·cm^2^, for new, and aged samples, respectively. As pointed above, this variation, could be related to errors, due to the subtraction of electrolyte resistance, especially relevant when working with highly conductive electrolytes and low resistive separator, as in this case. Additionally, the apparent similarity of corresponded ohmic resistance obtained for all samples, can be also explained, considering that ohmic resistance is contributed by several resistances: Electrode materials, electric contact, electrolyte, etc. [28], and for EIS-method, the same electrodes and electrolyte conditions were used.

When compared with DC method, more stable and reproducible values of ohmic resistance (and thus area resistance (AR)) were obtained by this EIS technique, which may be considered more appropriate for electrochemical characterization of low resistance components. 

### 3.3. Zero-Gap EIS

**Figure 15:**
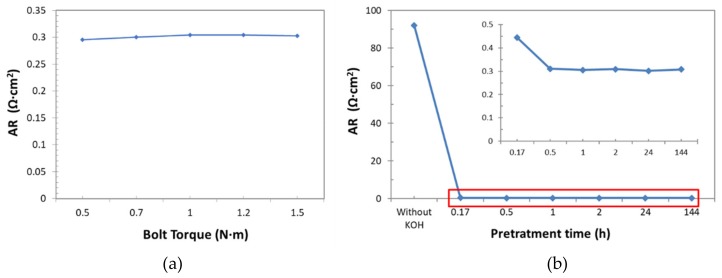
Influence of Zero-Gap Method parameter over area resistance (AR) determination: (**a**) Bolt Torque (**b**) Pre-treatment time.

For this approach, the influence of the bolt torque of set-up cell, as well as of the pre-treatment of samples on the resistance measurements (Figure 15a) were firstly analyzed. 

To evaluate the right bolt torque to apply to the sample, a new sample of Zirfon^®^ was immerged in KOH (30 wt%) for 24 h, then introduced into the cell, for the AR determination (as described in Section 2.3). The bolt torque values adopted, ranged from 0.5 to 1.5 N·m. Results reported in Figure 14a indicated that there was not a significant effect on AR observed in this examined bolt torque range: Only a low increase in the resistance was observed from 0.5 up to 1 N·m, but then, AR values were practically the same. Value of 1 N·m was selected as nominal bolt torque.

To determine the suitable pre-treatment time, new samples were immerged in KOH (30 wt%) at ambient temperature for different times: 0h, 0.17 h, 0.5 h, 1 h, 2 h, 24 h, and 144 h. Figure 15b shows that, unexpectedly, low pre-treatment times were enough to fill the diaphragm channels. In fact, after 30 minutes the AR values did not change significantly. So, in order to guarantee a complete filling of channels a pre-treatment time of 1 h was considered suitable for AR determination.

**Figure 16:**
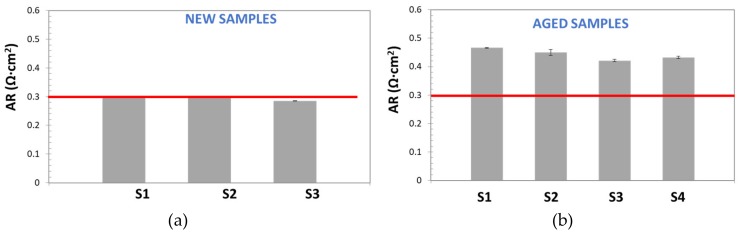
Area resistance values calculated by Zero-Gap EIS method for: (**a**) New diaphragm samples (**b**) Used diaphragms.

After fixing the previous operational aspects, the AR experiments were carried out (Figure 16). Also, in this method, inductance correction was carried out, as previously described. The results demonstrated that this technique showed higher repeatability, precision, and accuracy than previous techniques, even than the EIS-based one. It is worth noting the low variation of standard deviation for both types of samples (aged and new ones). 

Regarding the new samples, AR determination reported very close values to reference one. This is a confirmation of accuracy of proposed method. For aged samples, a reproducible response was also obtained. In addition, in this case, AR was higher (between 40% and 50% higher) than reference value for all analyzed samples. The increase in resistance of diaphragms after long operation times can be expected and it is in good agreement with the reduction of the cell performance with time operation, discussed above. 

### 3.4. Comparison between Techniques

In Table 2, the results of the analysis of all three studied techniques are shown. In particular, the values of resistance, standard deviation, coefficient variation, error, ionic conductivity, and tortuosity, are reported. 

If the resistance is concerned, we must notice that its value is mostly determined by material and geometry. Being the same material for all cases, resistance values strongly depend on sample geometry, which is defined by system architecture. Therefore, in order to correctly compare the different techniques, a proportional resistance concept (as area resistance) could be more suitable, because it does not depend on geometry. However, since precision is analyzed in terms of standard deviation and coefficient variation, which depend on resistance, to a more complete analysis, in the present work, resistance average values are reported.

Taking this into account, from Table 2 it is seen that DC technique is the least precise, because it reported a higher variability of resistance measurements, as confirmed by the high values of coefficient variation: 32% and 41.7%. These results suggest that experimental resistance measurements varied in a wide range, as shown in Figure 10. The low precision of this technique was also observed by other authors [13]. Despite this, DC technique is widely used for membrane resistance determination because its simplicity and low technique requirements. In fact, although in this work a potentiostat/galvanostat was used for electric measurements, this technique can be also undertaken by using a power source and a common voltmeter, as reported in the literature [29].

In contrast, EIS techniques showed very low CV values, confirming that alternating the current techniques are more precise than DC techniques [7]. Among them, EIS-Zero Gap technique reported values as low as 0.33%, demonstrating that it is the most repeatable method. The difference between both EIS techniques is related to the presence of an electrolyte volume between the electrodes and separator, which also has an ohmic contribution, thus increasing the error of the EIS-method.

**Figure 17:**
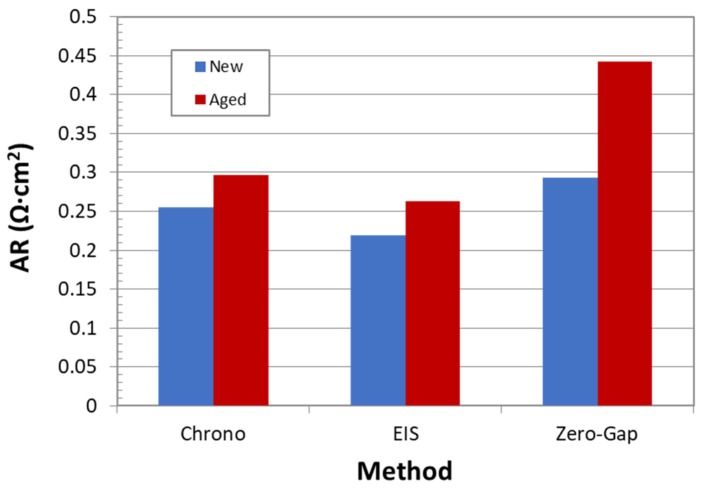
Comparison of three studied methods in terms of Area Resistance.

If accuracy data are considered, they were evaluated by contrasting experimental values of area resistance with the theoretical one, supplied by the manufacturer. Figure 17 reports values of area resistance for new and aged samples for all three studied techniques. The comparison must be mainly done with respect to new samples, because it is very difficult to find comparable references for aged samples.

In this case, the best results also correspond to the Zero-Gap EIS method. In fact, the calculated area resistance value is practically equal to the reported one (0.3 Ω·cm). Unlike the expected behavior, DC technique showed a higher accuracy than EIS method. In the opinion of the authors, considering the high variability of DC methods, the result obtained by this technique are not reliable. Nevertheless, even if the EIS method is an accurate technique, the obtained result is far from the reference one. This poor performance can probably be related to ohmic contribution of electrolyte and subtraction calculations. This can be also confirmed by the fact that even aged samples resistance, determined by this technique, is much lower than the reference value for new separators. 

Furthermore, although no aged samples references were found, the convenience of the EIS-Zero Gap method for aged samples can be evaluated in terms of ohmic contribution with respect to the total ohmic overpotential of the cell. According to the literature, the ohmic over-potential can represent up to 20% of the overall voltage in an alkaline electrolysis cell when the current density is 400 mA/cm^2^, being the energy loss due to bubble formation on the electrodes the major contribution to the total over-potentials (more than 50%), and the separator about 15% [5,30]. So, the diaphragm contributes up to 3% of the overall cell potential at high current densities. As shown in Figure 17, an increase in the AR of 50% is observed in the aged diaphragm, so the contribution of the separator in the ohmic overpotential could increase up to 4–5%. On the other hand, according to Figure 8, an increase in potential of approximately 4% occurs after 130 h of operation, so it seems that there is a direct relationship between the increase in the cell potential and the resistance measured with the EIS zero-gap technique.

Finally, besides precision and accuracy, in Table 2 conductivity and tortuosity are also reported. Ionic conductivity corresponds to conductivity of electrolyte inside the separator channels. On the other hand, tortuosity gives information about irregularity and sinuosity of channels inside the separator. The values in the range of 1.5–1.9 were obtained in this study for new samples. The ionic conductivity and tortuosity agree with the data reported in literature [11,31]. Thus, it can be concluded that a precise approach to determining the resistance contribution of diaphragms can be also useful for a more complete material characterization, including geometrical and physical-chemical parameters. Then, the choice of a suitable approach becomes critical. Among the analyzed methodologies in this study, the proposed Zero Gap-EIS method was revealed to be the most convenient one in terms of simplicity, accuracy, and precision.

## 4. Conclusions

A simple and repeatable methodology, based on Electrochemical Impedance Spectroscopy, was presented in this work, and its application to diaphragm characterization was studied. Furthermore, the application of this method was compared with two, well-known methodologies, commonly used in the literature: DC-based technique and an EIS-based one. For comparison purposes, a commercial diaphragm normally used in AWE, Zirfon^®^, was selected as reference material. 

DC-based methodology showed low repeatability, and poor accuracy and precision for standard deviation values, which varied in the range of 30–40%. Experimental deviation agreed with the observations by other authors. Although it is a simple technique, due to the poor results, it is not considered a suitable solution for determining the diaphragm resistivity in AWE.

On the other hand, the EIS-based technique reported good accuracy, but a lower precision than expected. Standard Deviation values close to 4–5% were calculated, confirming the good repeatability of this technique. Nevertheless, the area resistance, obtained by this approach, was the lowest, far away from the reference value of the commercial material. The cause of this strong difference can be found in the contribution to the ohmic resistance of the electrolyte filling the anodic and cathodic compartment. Being the resistance of diaphragm at a low value, the subtraction of the resistance of a substantial electrolyte volume can increase the error due to the calculations. These results suggested that, despite the convenience of using alternating current measurements instead of direct current ones, a not-subtracting approach can be preferred for high porosity diaphragms, in strong concentrated media, in order to reduce errors from calculations. 

In relation to the Zero Gap-EIS Method proposed in this work, the most accurate and precise results were obtained by this approach. The good performance of this methodology was due to two aspects: Use of EIS technique favored a repeatable experimental response; and the elimination of the correction with the electrolyte resistance, which reduced the total error. As consequence, very low standard deviation values (in the range of 1%) were obtained, and the area resistance calculated for Zirfon^®^ was almost the same than reported by the manufacturer. 

In addition, in order to ascertain the influence of operation time over the performance of the diaphragm, the aged diaphragm samples were also studied. For all methods, a higher resistance was calculated for aged samples with respect to the new ones. The increasing resistance was related to the deposition of metal compounds on the surface of diaphragm, which could block the channels, and limit the OH^−^ ions transport.

Finally, it was confirmed that, by using the described methodologies, other physical-chemical properties (tortuosity and ionic conductivity) of diaphragms can be determined, allowing a more complete characterization of these components. Accordingly, choosing a suitable approach for diaphragm resistance determination becomes critical. 

In the present study, the proposed Zero Gap-EIS methodology showed to be the optimal option. Authors expect that this simple and economical approach could contribute to propel the future research on new membranes and diaphragms materials for Alkaline Water Electrolysis. 

## Figures and Tables

**Table 1:** Zirfon Perl UTP 500^®^ mean features [19].

Property	Value
Weight Density (g·cm^−3^)	1 ± 0.2
Thickness (µm)	500 ± 50
Maximum Temperature (°C, continuous operation)	110
Thermal stability at 100 °C (% shrinkage)	<1.5
Shelf life (months, unopened packaged)	12
Porosity (%)	50 ± 10
Bubble point (bar)	2 ± 1
Pore size (µm)	0.15 ± 0.05
Gas Permeability (l/min·cm^2^ at 5 bar)	4 ± 1
Ionic Resistance (Ω·cm^2^ at 5 bar)	<0.3
Maximum current density (kA/m^2^)	20
Maximum electrolyte strength (wt% KOH or NaOH)	30
Lifetime expectancy (years, under normal operating conditions)	>5

**Table 2:** Resistance and error values for new and aged diaphragm samples for accuracy and precision comparison of studied methods. Also, Ionic Conductivity, and Tortuosity values are reported.

Method	Sample	R (Ω)	σ	CV	κ_D_ (S⋅cm^−1^)	τ	Area Resistance (Ω⋅cm^2^)	Error (%)
Chrono	Aged	0.062	±0.02015	32%	0.168	1.92	0.2967	-
	New	0.0515	±0.0215	41.7%	0.2042	1.55	0.255	15%
EIS	Aged	0.02574	±0.0009	3.44%	0.190	1.71	0.26253	-
	New	0.0215	±0.00121	5.64%	0.228	1.42	0.2188	23%
EIS-Zero Gap	Aged	0.2209	±0.0025	1.16%	0.113	2.84	0.442	-
	New	0.1467	±0.0005	0.33%	0.170	1.89	0.2935	3.2%
